# PAMs inhibits monoamine oxidase a activity and reduces glioma tumor growth, a potential adjuvant treatment for glioma

**DOI:** 10.1186/s12906-020-03041-z

**Published:** 2020-08-15

**Authors:** Pei-Chuan Li, Shih-Yi Chen, Danzhou Xiangfei, Canquan Mao, Chieh-His Wu, Jean Chen Shih

**Affiliations:** 1 Department of Pharmacology and Pharmaceutical Sciences, School of Pharmacy, University of Southern California, Rm. 518, 1985 Zonal Ave, Los Angeles, CA 90089 USA; 2 USC-Taiwan Center for Translational Research, School of Pharmacy, University of Southern California, Los Angeles, CA 90089 USA; 3 School of Pharmacy, Taipei Medical University, Taipei, 110 Taiwan; 4 Institute of Yunnan Folk Medicine, Kunming, 650202 China; 5 School of Life Sciences and Engineering, Southwest Jiaotong University, Chengdu, 610031 China; 6 Department of Cell and Neurobiology, Keck School of Medicine, University of Southern California, Los Angeles, CA 90089 USA; 7 Norris Comprehensive Cancer Center, Keck School of Medicine, University of Southern California, Los Angeles, CA 90089 USA

**Keywords:** Natural plant antimicrobial solution (PAMs), Monoamine oxidase a (MAO a), Temozolomide (TMZ), Glioblastoma, Glioma, Reduce glioma growth

## Abstract

**Background:**

Monoamine oxidase (MAO) A catalyzes oxidative deamination of monoamine neurotransmitters and dietary amines and regulates brain development and functions. Recently, we showed that MAO A mediates the progression and migration of glioma and MAO A inhibitors reduce glioma cell growth. Glioblastoma (GBM) is a common and most malignant brain tumor which is difficult to treat. Temozolomide (TMZ) is the current standard chemotherapy for glioma, but tumors usually become resistant and recur. So far, no effective therapy for TMZ-resistant glioma is available. Natural plant antimicrobial solution (PAMs) is a Chinese herbal medicine which has been used for decades without toxicity and has multiple medical functions including anti- inflammatory effects. Here, we report the effects of PAMs on glioblastoma growth.

**Methods:**

The growth of TMZ -sensitive (U251S),-resistant (U251R) human glioma cells, and mouse glioma cell line GL-26 were assessed by MTS colorimetric assay, colony formation, and cell migration assays.

Male C57BL/6 mice were implanted subcutaneously or intracranial with luciferase-positive mouse glioma GL-26 cells and treated with vehicle; MAO A inhibitor clorgyline (10 mg/kg); TMZ (1 mg/kg); PAMs (48 mg/kg) alone or in combination with TMZ (1 mg/kg) for 14 days. At the end of the treatment, mice were sacrificed, MAO A catalytic activity in tumors was measured, and tumor sizes were determined by imaging and weight.

**Results:**

These results show that PAMs inhibits MAO A catalytic activity in all three glioma cell lines studied U251S, U251R, and GL-26. PAMs reduced glioma growth and has greater effects in combination with low dose of TMZ than PAMS or TMZ alone in all three cell lines as shown by MTS, colony formation, and cell migration assays. Using the subcutaneous or intracranial GL-26 glioma mouse model, PAMs reduced the tumor growth and MAO A activity, similar to the MAO A inhibitor clorgyline. Combining PAMs with non-toxic dose TMZ increased survival to a greater extent than those of PAMs or TMZ alone.

**Conclusions:**

This is the first study which suggests that PAMs alone or co-administration with low doses of TMZ may be a potential adjuvant to reduce the toxicity of TMZ and to abrogate drug resistance for the effective treatment of glioma.

## Background

It has been shown that monoamine oxidase A (MAO A) is overexpressed in prostate cancer [1], glioma [2] and Hodgkin lymphoma [3], inhibiting MAO A reduces tumorigenesis and metastasis. MAO A is located in the outer membrane of mitochondria, catalyzes oxidative deamination of neurotransmitters or dietary amines and produces H2O2 [4, 5]. MAO A inhibitors have been used for decades for the treatment of neuropsychiatric disorders [6].

Glioblastoma (GBM) is the most aggressive form of primary brain tumors with a median survival of 14 months from the time of diagnosis [7, 8]. The standard treatments of newly diagnosed glioblastoma (GBM) are surgery, radiation therapy administered concurrently with oral temozolomide (TMZ), and six cycles of adjuvant TMZ therapy [9]. Unfortunately, less than 10% of the 5-year survival rate accounts for high-grade glioma patients treated with the standard radiotherapy and adjuvant chemotherapy [10]. Almost half of all gliomas are neither TMZ responsive nor susceptible to radiotherapy. Further, increasing the dose of TMZ, a DNA alkylating agent, will increase the toxicity to the bone marrow [11]. Thus, better treatment for GBM is urgently needed. Our previous study showed that MAO A inhibitor clorgyline combined with non-toxic dose TMZ reduced the tumor growth and prolong the survival in animal model [2].

Plant Antimicrobial Solution (PAMs) has anti- inflammation effect, was used for wound infection and festering, cell necrosis, dry gangrene and blood circulation obstacles [12] and was approved to be used as a hospital preparation by China Yunnan Food and Drug Administration.

PAMs is a mixture of Chinese herbal medicine consists of plants, including *Carthamus tinctorius*, *Cymbopogon distans*, *Lithospermum erythrorhizon*, and *Solanum indicum*, and *Blumea balsamifera*. we have identified two active compounds from PAMs including *Hydroxysafflor yellow A* (HSYA) in *Carthamus tinctorius* and *Allantoin* in *Cymbopogon distans*. HSYA exhibited anti-cancer and anti-inflammation function [12] while Allantoin has wound healing function [13]. Recently, we found Shikonia from *Lithospermum erythrorhizon* inhibited MAO A catalytic activity (unpublished data). Using network pharmacology from three database (TCMSP, Batman and YaTCM), we identified 158 compounds from the herb plants present in PAMs which may be the active components. This information will help us purify and identify additional active ingredients in PAMs by HPLC, GC, and Mass Spectroscopy.

Previous studies showed that PAMs inhibits the TNF- α/IFN-γ-induced inflammatory cytokines production in HaCaT cells and ameliorates imiquimod- induced psoriasis-like skin inflammation in vivo through inhibiting the translocation of p65 in NF- κB signaling pathways [12]. Our previous studies showed that treatment with MAO A inhibitor increased TNF-α positive cell population in tumors from glioma animal model [2]. Recently, it has been reported that treatment with MAO A inhibitor reduced the expression of the oncogene NF-κB in prostate cancer [14]. Taken together, this data suggests that MAO A inhibitors regulate the inflammatory response to suppress tumor progression. These findings led us to study if PAMs may have similar properties as a MAO A inhibitor.

## Methods

### Preparation of PAMs

PAMs was obtained from the Institute of Yunnan Folk Medicine and produced by Yunnan Puer Danzhou Pharmaceutical Co., Ltd. (Yunnan Province, P.R. China) [12]. Briefly, 5 ml medicinal plants mixture PAMs including *Carthamus tinctorius, Cymbopogon distans, Lithospermum erythrorhizon, Solanum indicum*, and *Blumea balsamifera*, was diluted with 5 ml H2O, concentrated by vacuum at 30 °C for 1–2 h. Another 5 ml PAMs and 5 ml H2O mixture were added, vacuumed again to 2 ml. The concentrated sample was lyophilized (− 80 °C) overnight to powder. The dried PAMs powder (40 mg) was re-dissolved in 1 ml 2% ethanol kept at − 20 °C until use.

### Cell culture

Human glioma cell lines U251S (TMZ sensitive) U251R (TMZ resistant), and mouse glioma cell line GL-26 with luciferase were provided by Dr. Florence Hofman at the University of Souther California; Glioma cell lines [15] were cultured in 10% fetal bovine serum in Dulbecco’s Modified Eagle’s Media (Life Technologies, Carlsbad, CA, USA) supplemented with 100 U/ml penicillin and 0.1 mg/ml streptomycin in a humidified incubator at 37 °C and 5% CO2.

### MAO a catalytic activity assay

MAO A catalytic activity was determined by radioassay as described previously [1, 3]. Human prostate cancer LNCaP cells and mouse glioma GL26 cells were used. Clorgyline (Sigma-Aldrich) was pre-incubated with cells at 37 °C for 20 min, then the substrate 1 mM ^14^C-5-hydroxytryptamine (5-HT) was added, incubated for 20 min at 37 °C. At the end of the incubation, the reaction product was extracted, and the radioactivity was determined by the scintillation counter (LS 6500, Beckman coulter, Inc., CA, USA).

### MTS assay

Glioma cells U251S, U251R, or GL-26 (5 × 10^3^ cells/well) were seeded in 96-well plates and treated with PAMs at various concentrations (0–150 μg/ml) for 48 h. Cell proliferation was determined by an MTS assay kit (Promega, WI, USA). Briefly, MTS reagent (20 μl/well) was added into each well and incubated for 4 h in the presence of 5% CO2 at 37 °C. The plate was gently agitated prior to colorimetric analysis, the reaction product of MTS was measured at 490 nm and quantified using a microplate reader Synergy HTX (Bio-Tek, Winooski, VT, USA). Data was plotted using GraphPad Prism (GraphPad Software, San Diego, CA, USA). For the combined treatment with TMZ (Sigma-Aldrich), 5 × 10^3^ glioma cells were seeded per well in triplicate and pre-treated with TMZ (15 μM) for 48 h followed by incubation of PAMs and TMZ for 48 h. All experiments were repeated at least three times.

### Colony forming assay

Glioma cells were seeded in 96-well plates, various concentrations of PAMs with or without TMZ (15 μM) were incubated for 48 h. After 48 h, cells were re-seeded 500 cells/per well with fresh medium in duplicate. Cells were incubated for an additional 8 to 10 days; colonies were visualized by staining with 1% methylene blue in methanol for 4 h and quantified. Colonies were enumerated from pictures of plates using software tools (open CFU).

### Cell migration assay

Glioma cells (1.2 × 10^6^ cells/ well) were seeded in 24-well plates for 24 h, then the monolayer was scratched with a new 200 μl pipette tip, fresh medium containing various concentrations of PAMs with our without 15 μM TMZ, were incubated for 24 h. Migrated cell numbers in the scratched region were visualized by staining with 1% methylene blue in methanol for 4 h and quantify by imaging. (Image J software, National Institute of Health, Bethesda, MD, USA.

### Animal models

Male 4- to 6-weeks-old C57BL/6 mice were purchased from Harlan (Indianapolis, IN), house in the animal research facility at University of Southern California (USC), fed with a normal diet. All animal protocols were approved by the Institutional Animal Care and Use Committee (IACUC No. 20212) of USC. Mice were sacrificed by carbon dioxide (CO2). For subcutaneous model, Xenograft tumors were established via subcutaneous inoculation of 5 × 10^3^ luciferase-positive mouse glioma GL-26 cells into the flank of the mice. Six days after the inoculation, mice were subcutaneously injected with clorgyline (10 mg/kg), PAMs (48 mg/kg) and vehicle respectively for 14 days. Subcutaneous injections were administered in the space between the skin and the underlying muscles over the shoulders.

For intracranial model: mice were treated with (1) PAMs (2) TMZ (1 mg/kg) alone, (3) TMZ and PAMs combination for 14 days. PAMs was injected daily intranasally (IN). TMZ was dissolved in water and administered by gavage for 10 days. Combination of TMZ and PAMs were given with the same schedule as each agent alone.

### Statistical analysis

All data was presented as the mean ± standard error (SE) values and analyzed using GraphPad Prism 6 (GraphPad Software, San Diego, CA, USA). T-test was performed for comparison with multiple groups. In brief, we analyzed the data using Prism (two samples and paired) by t-test analysis. A *p*-value of *p* < 0.05 was considered statistically significant.

## Results

### PAMs inhibits MAO a catalytic activity in glioma cells

**Fig. 1:**
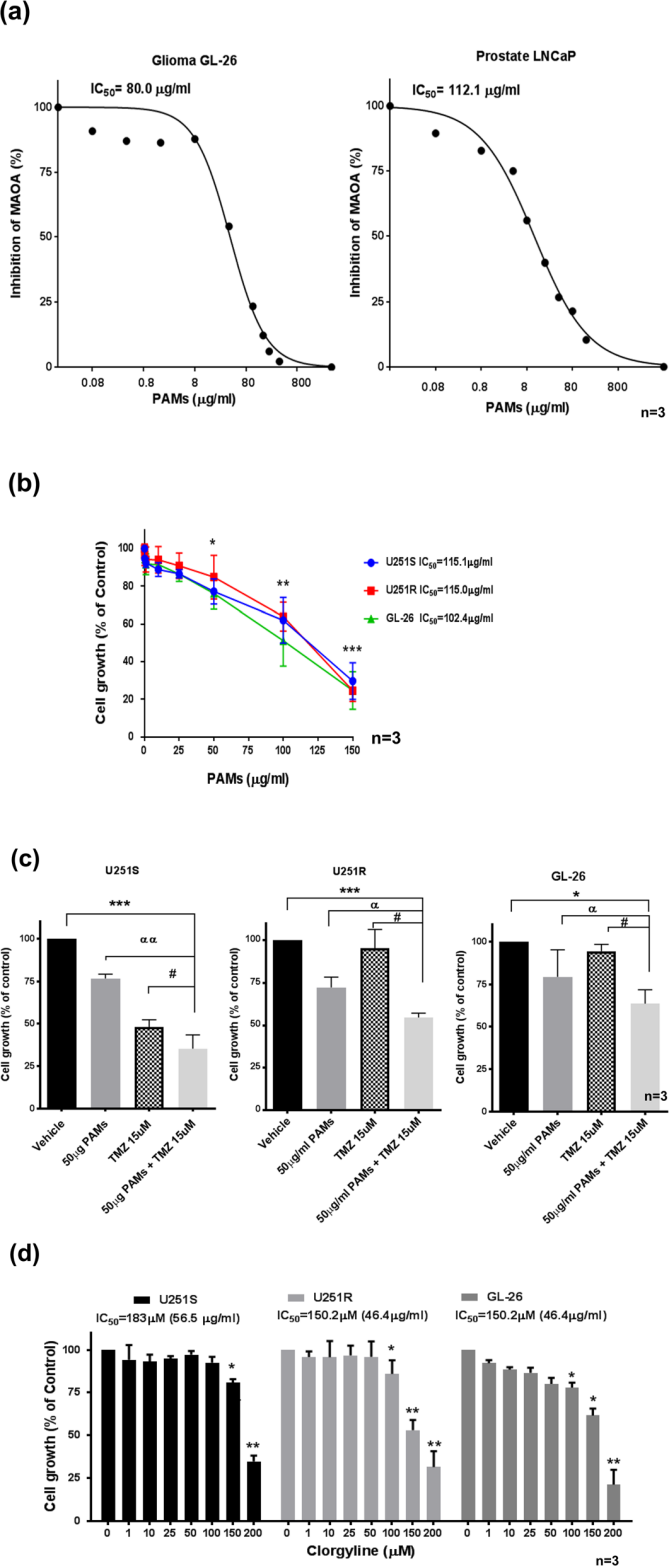
PAMs reduced MAO A activity and cell growth in glioma cells in vitro. **a** PAMs inhibited MAO A catalytic activity. The effect of PAMs on MAO A catalytic activity was determined in mouse glioma GL-26 and human prostate LNCaP cells. PAMs at appropriate concentrations were pre-incubated at 37 °C for 20 min, then the substrate serotonin (5HT) was added for MAO A catalytic activity assay, see text for detail. 100% of MAO A catalytic activity was 36.65 or 29.86 nmol/20 min/mg in prostate cancer or glioma respectively. **b** Effect of PAMs. on cell growth in human glioma TMZ-sensitive (U251S), TMZ-resistant (U251R) and mouse GL- 26 cells as determined by MTS assay. 5 × 10^3^ cells were seeded in each well, incubated with PAMs at (0-150 μg/ml) for 48 h, MTS assay was then performed. **c** PAMs in combination with low dose TMZ reduced cell growth in U251S, U251R, and GL-26 cells. Cells were treated with PAMs (50 μg/ml), TMZ (15 μM), PAMs (50 μg/ml) + TMZ (15 μM) for 48 h, MTS assay was then performance. Data represent the mean ± SEM. The *p* value was calculated by t-test. *p < 0.05, **p < 0.01, ***p < 0.001 compared to control (0 μg). #p < 0.05, PAMs (50 μg/ml) + TMZ (15 μM) compared to TMZ alone. **d** Effect of MAO A inhibitor clorgyline on cell growth. Cells (U251S, U251R, and GL-26) were seeded and incubated with clorgyline (0-200 μM). The IC50 of clorgyline is 56.5, 46.4, and 46.4 μg/ml for U251S, U251R, and GL-26 cells. Experiments were performed in triplicate and repeated three times with similar results (*n* = 3)

The effect of PAMs on the inhibition of MAO A catalytic activity was determined in mouse glioma GL-26 cells [2] and human prostate LNCaP cells which have been reported to express high MAO A activity [1]. Our results showed that IC50 of PAMs is 80.0 μg/ml in GL-26 cells and 112.1 μg/ml in LNCaP cells (Fig. 1a). These results indicate that PAMs inhibits MAO A catalytic activity in both glioma and prostate cancer cell lines with almost similar potency.

### PAMs inhibits the growth of human TMZ-sensitive U251S, TMZ-resistant U251R cells and mouse glioma GL-26 cells

To determine the effects of PAMs on the cell viability on glioma. Human glioma cell lines U251S, U251R, and mouse glioma GL-26 cells were used. In this study, we treated the cell with PAMs at 24 h and 48 h, with different concentrations. We found 48 h was the optimum condition; thus, we showed the data at 48 h. Cells were incubated with various concentrations of PAMs (0.1 to 150 μg/ml) for 48 h. Cell viability was determined by MTS assay. PAMs showed dose-dependent inhibition with 50% inhibitory concentrations (IC50) of 115.1, 115.0, and 102.4 μg/ml for U251S, U251R, and GL26 cells, respectively (Fig. 1b).

Further, the effect of PAMs in combination with low dose 15 μM TMZ on inhibiting the growth of glioma cell lines (U251S, U251R, GL-26) was investigated. PAMs (50 μg/ml) alone inhibited the growth by 24, 28, and 21% in U251S, U251R, and GL26 cells, respectively. Combining low dose TMZ with PAMs reduced cell growth by 65%, 46 and 36% in U251S, U251R, and GL-26 cells, respectively, they were more effective compared to PAMs alone (αα, *p* < 0.01, U251S α, *p* < 0.05, GL-26), TMZ alone (#, p < 0.05,U251R) or vehicle (***, *p* < 0.001, U251S and U251R; *, p < 0.05, GL-26) (Fig. 1c).

We also evaluated the effect of clorgyline on human TMZ-sensitive U251S or TMZ-resistant U251R cells, and mouse glioma GL-26 cells for comparison. Clorgyline suppressed the growth of all three cell lines similarly, IC50 = 56.5, and 46.4 μg/ml for U251S and R cells, 46.4 μg/ml for GL26 cells (Fig. 1d).

**Fig. 2:**
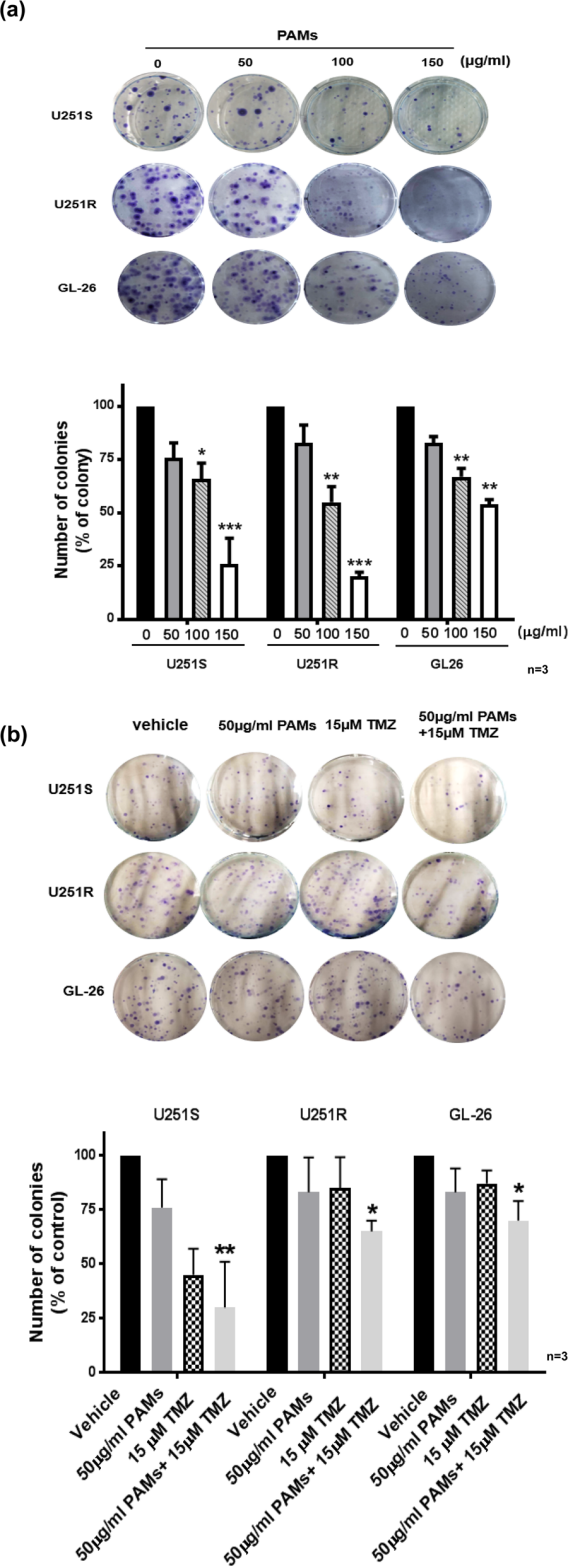
PAMs reduced cancer cell growth in human glioma TMZ-sensitive (U251S), TMZ-resistant (U251R) and mouse GL-26 cells as determined by colony formation assay. **a** PAMs reduced cell growth as determined by colony formation in glioma cell lines (U251S, U251R and GL-26). 500 cells were seeded in each well, treated with PAMs for 48 h, incubated for additional 8–10 days, cells were fed every two days. **b** Cells were treated by PAMs (50 μg/ml), TMZ (15 μM), or PAMs (50 μg/ml) + TMZ (15 μM). Combination of PAMs with low dose TMZ significantly inhibited cell growth as shown by colony formation assay. Data represent the mean ± SEM. The *p*-value was calculated by t-test. *p < 0.05, **p < 0.01, compared to untreated control (0 μg/ml). The untreated control group was taken as 100%. After combined treatment of PAMs and TMZ, percentage of colonies were 30, 60, and 60 (colony size> 100 mm) in U251S, U251R, and GL-26 cells respectively. Experiments were performed in triplicate and repeated three times with similar results (*n* = 3)

Figure 2a shows the inhibitory effect of PAMs on the growth of U251S, U251R, and GL-26 cells using colony formation assay. The quantitative data showed that PAMs suppresses cell growth and colony formation in a dose-dependent manner (Fig. 2a). 50 μg/ml of PAMs reduced the growth by 24, 17, and 17% in U251S, U251R, and GL-26 glioma cells respectively (Fig. 2a). PAMs showed its effect in a dose dependent manner. With colony reduction efficacy of 34, 45, 33% in U251S, U251R, and GL-26 at 100 μg/ml, and 74, 80, 46% in U251S, U251R, and GL-26 at 150 μg/ml (Fig. 2a).

PAMs (50 μg/ml) in combination with low dose TMZ (15uM,2.91μg/ml) suppressed colony formation by 70, 35, 30% in U251S, U251R, and GL-26 cells, respectively (**, *p* < 0.01 in U251S; *, *p* < 0.05 in U251R and GL-26) (Fig. 2b,). 15uM TMZ inhibited colony formation by 55, 15, 13% in U251S, U251R, and GL-26 cells. This data indicates that PAMs in combination with TMZ reduces cell growth in vitro more effectively than each compound alone.

### PAMs inhibits the migration of human TMZ-sensitive U251S, TMZ-resistant U251R or mouse glioma GL-26 cells

**Fig. 3:**
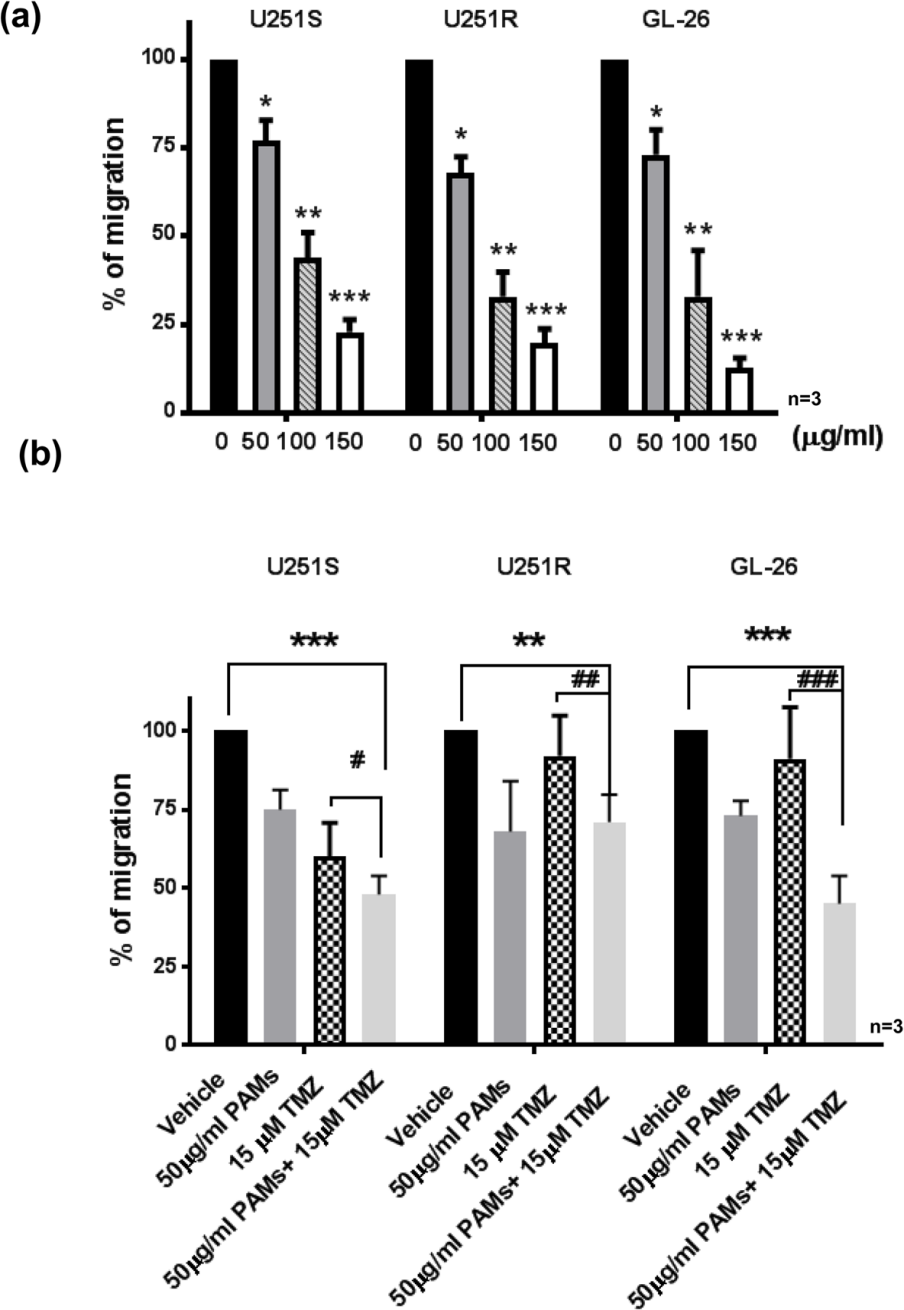
PAMs suppressed the cells migration in human glioma TMZ-sensitive (U251S), TMZ- resistant (U251R) and mouse GL-26 cells cancer either alone or in combination with TMZ We determine the migration of glioma cells by treating with (**a**) PAMs alone, 1.2 × 10^6^ cells were seed in 24 well and incubated PAMs at various concentrations (0, 50, 100, 150 μg/ml) for 24 h or (**a**) PAMs in combination with low dose of TMZ. Cells were treated by PAMs (50 μg/ml), TMZ (15 μM), or PAMs (50 μg/ml) + TMZ (15 μM) and then cell numbers were counted and quantified. Data represent the mean ± SEM. The *p*-value was calculated by t-test. (*p < 0.05, **p < 0.01, ***p < 0.001 compared to untreated control. #*p* < 0.05, ##*p* < 0.01, ###*p* < 0.001 compared to TMZ alone). The untreated control groups were taken as 100%. Experiments were performed in triplicate and repeated three times with similar results (*n* = 3)

The inhibitory effect of PAMs on the migration of glioma cells was studied in all three cell lines. Glioma cells were treated with various concentrations (0, 50, 100, or 150 μg/ml) of PAMs for 24 h. As shown in Fig. 3a, 50 μg of PAMs significantly inhibited migration by 25% (*, *p* < 0.05), 32% (*, p < 0.05), 27% (*, p < 0.05) in U251S, U251R, and GL-26 cells, respectively (Fig. 3a). Further increase of PAMs concentrations show that PAMs suppresses the migration of glioma cells in a dose-dependent manner. Higher dose of PAMs (100 or 150 μg/ml) was more effective, with inhibition of 56% (**, *p* < 0.001),67% (**, p < 0.001), 67% (**, p < 0.001) in U251S, U251R, and GL-26 at 100 μg/ml; 77% (***, p < 0.001), 80% (***, *p* < 0.001), 87% (***, p < 0.001) in U251S, U251R, and GL-26, at 150 μg/ml. Thus, PAMs significantly reduced cell migration in all glioma cell lines we studied (Fig. 2a).

Next, the effect of combining PAMs with low dose TMZ was assessed. Figure 3b shows that 50 μg PAMs in combination with 15 μM TMZ significantly inhibited migration compared to vehicle by 52% (***, p < 0.001), 29% (**, *p* < 0.01), 55% (***, p < 0.001) in U251S, U251R, and GL-26 cells, respectively. 15uM TMZ alone inhibited cell migration by 40, 8, 9% in U251S, U251R, and GL-26 cells. When compared with TMZ alone, PAMs in combination with TMZ inhibited more effectively: by 12% (#, *p* < 0.05), 21% (##, p < 0.01), 46% (###, p < 0.001) in U251S, U251R, and GL-26 cells (Fig. 3b), respectively. This finding suggests PAMs in combination with TMZ show more than additive effect compared with each compound alone in the migration assay (Fig. 3b).

### PAMs reduces the growth of tumors derived from GL-26 glioma cells implanted subcutaneously in C57BL/6 mice

**Fig. 4:**
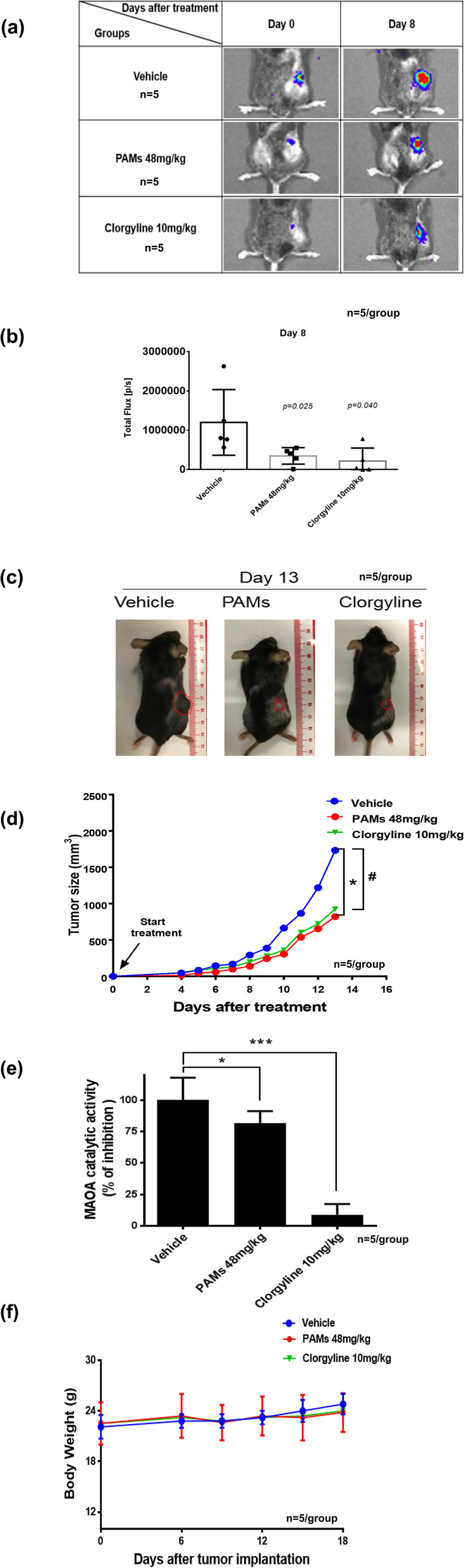
PAMs reduced tumor growth, similar to MAO A inhibitor clorgyline in subcutaneous mouse glioma C57BL/6 mouse model. Mice were implanted subcutaneously with 5 × 10^3^ luciferase-positive mouse glioma GL-26 cells, then were treated with MAO A inhibitor clorgyline (10 mg/kg), PAMs (48 mg/kg), and vehicle for 14 days. **a** Tumor sizes were reduced by PAMs and clorgyline at day 8 after the implantation of GL-26 cells in C57BL/6 mice as shown by images. Representative images of tumor size reduced by PAMs or clorgyline on day 0 and 8 after treatment were shown. **b** Qualitative representation of bioluminescence imaging conducted at day 8. **c** PAMs and clorgyline reduced tumor size at day 13 in subcutaneously implanted GL-26 cells in C57BL/6 mice. **d** PAMs and clorgyline reduced tumor size compared to vehicle. **e** MAO A catalytic activity was reduced by PAMs and clorgyline. **f** No change in body weight was observed for all three groups, i.e. PAMs, clorgyline, and vehicle. Each group *n* = 5. Data represent the mean ± SEM. The *p*-value was calculated by t-test. *p < 0.05, PAMs group compared to vehicle, #*p* < 0.05 clorgyline compared to vehicle

To ascertain the in vivo effect of PAMs on tumor growth, murine glioma GL-26 cells were subcutaneously implanted in C57BL/6 mice. Six days post implantation (i.e. day 0 for drug treatment), mice were imaged and treated with PAMs (48 mg/kg), clorgyline (10 mg/kg) or vehicle. All treatments were administered subcutaneously daily for 14 days. Tumor size was measured by imaging and the imaging data shows decreased tumor size after treating with PAMs or clorgyline (Fig. 4a and b) and by the ellipsoid volume formula (π/6 x L x W x H and 1/2 x L x W x H) (Fig. 4c and d). PAMs reduced tumor size as determined by both methods on day 13 (47%) (Fig. 4d). As shown in Fig. 4d, both PAMs and clorgyline reduced tumor growth significantly and similarly (day 13, *, *p* < 0.05 PAMs group compared to vehicle, #, *p* < 0.05 clorgyline compared to vehicle). These data indicate that PAMs reduces tumor growth in a glioma mouse model. Similar results were obtained in mice treated with PAMs or clorgyline, i.e. tumor growth reduced by 53% (Fig. 4d). PAMs or clorgyline inhibited MAO A catalytic activity by 25% (*, *p* < 0.05, Fig. 4e) and 90% (***, *p* < 0.001, Fig. 4e) respectively. No change in body weight occurred when mice were treated with PAMs, clorgyline or vehicle (Fig. 4f). This study shows that PAMs inhibits MAO A activity and glioma cell growth without toxicity.

### The effect of PAMs on the growth of tumors derived from GL-26 glioma cells intracranially implanted in C57BL/6 mice

**Fig. 5:**
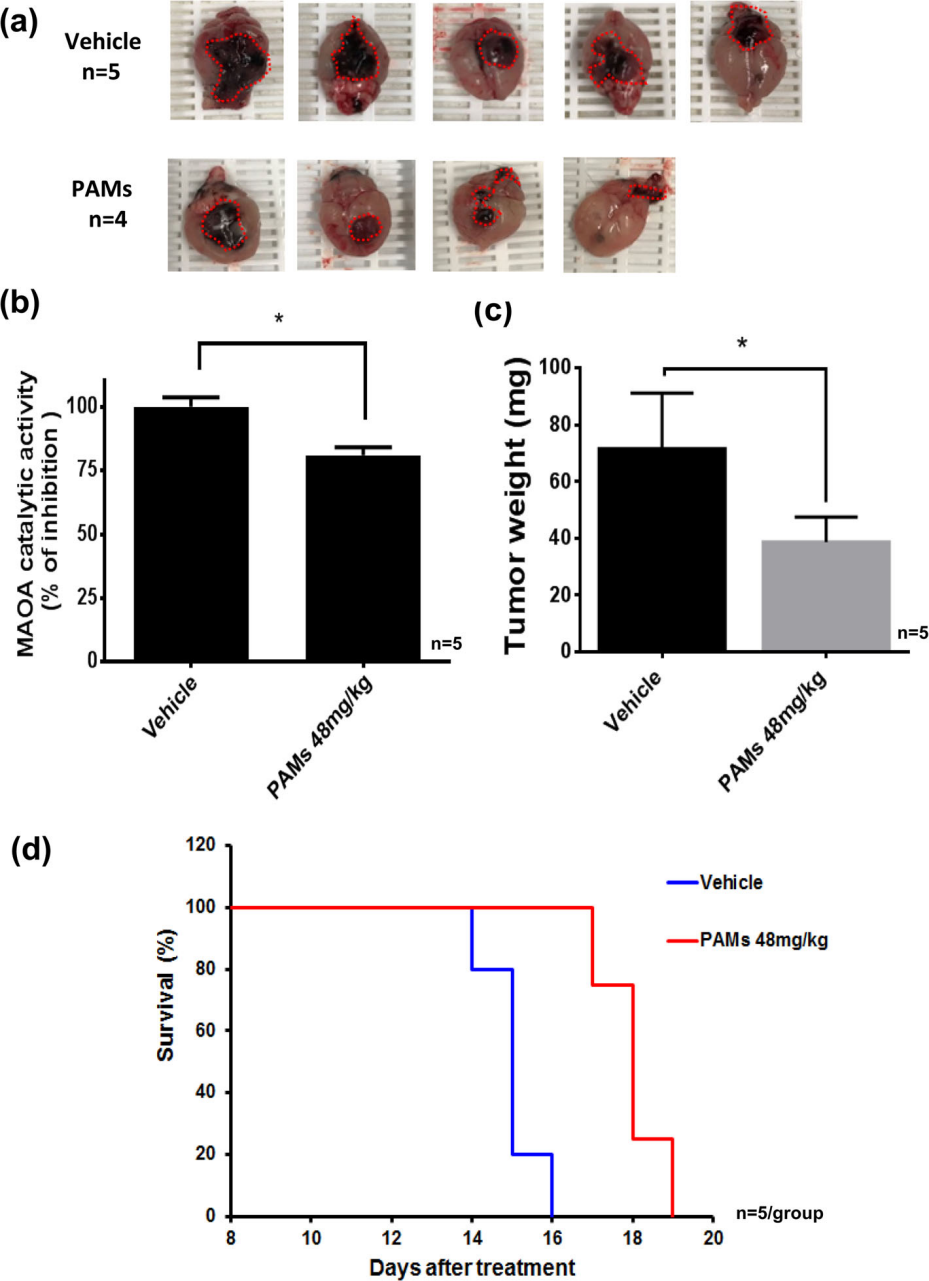
PAMs reduced tumor growth and increased survival in intracranial mouse GL-26 glioma C57BL/6 mouse model. Mice were implanted intracranial with mouse glioma GL-26 cells and treated with PAMs (48 mg/kg) or vehicle for 14 days. PAMs treated mice (**a**) reduced tumor size, **b** reduced tumor weight, and **c** inhibited MAO A catalytic activity in tumor compared to vehicle control. PAMs treated mice also had (**d**) increased survival compared to vehicle control. Each group *n* = 5

Mice were intracranially implanted with GL-26 glioma cells having luciferase activity. Six days later, mice were treated with PAMs (48 mg/kg) daily intranasally for 14 days. PAMs reduced the brain tumor size (Fig. 5a) and tumor weight compared to vehicle (* *p* < 0.05, Fig. 5b). MAO A activity was significantly reduced in PAMs treated brain tumor (25%) compared to the vehicle treated (*, p < 0.05, Fig. 5c).

**Fig. 6:**
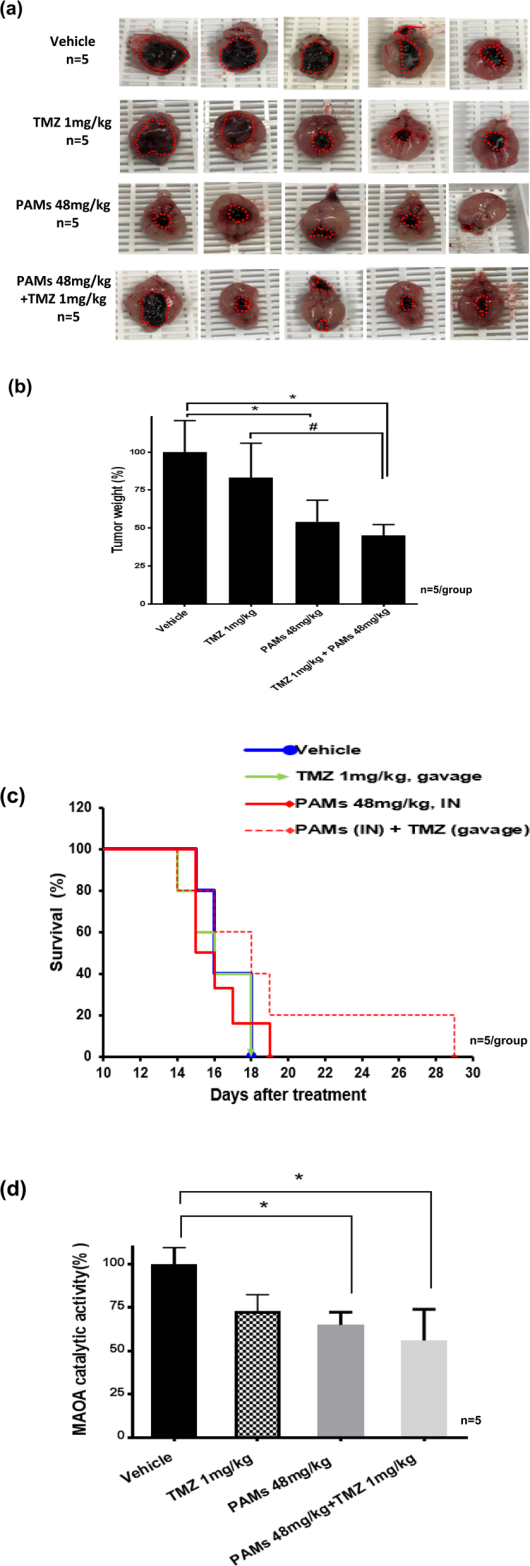
PAMs alone or in combination with TMZ increased survival in intracranial glioma C57BL/6 mouse model. Mice were implanted intracranially with mouse glioma GL-26 cells and treated with vehicle, and PAMs (48 mg/kg) by daily intranasal (IN) for 14 days, or TMZ (1 mg/kg) alone by oral gavage for 10 days, and in combination with PAMs and TMZ. **a** PAMs alone or in combination with TMZ reduced tumor growth in intracranial glioma mouse model. Mice with glioma intracranial tumor xenografts were treated by PAMs (48 mg/kg), TMZ (1 mg/kg), PAMs (48 mg/kg) + TMZ (1 mg/kg), and vehicle. Each group n = 5. **b** PAMs and PAMs (48 mg/kg) + TMZ (1 mg/kg) significantly reduced the tumor weight. **c** PAMs and PAMs (48 mg/kg) + TMZ (1 mg/kg) increased survival compared to vehicle or TMZ group. **d** PAMs or PAMs (48 mg/kg) + TMZ (1 mg/kg) suppressed MAO A catalytic activity compared to vehicle. Data represent the mean ± SEM. The *p-*value was calculated by t-test. *p < 0.05, PAMs group compared to vehicle

PAMs significantly increased survival (19 days) compared to vehicle (16 days) (Fig. 5d). The current standard chemotherapeutic for glioma is TMZ, whose efficacy is accompanied by toxicity and the onset of resistance. This result suggests that PAMs may be used to treat glioma without toxicity. Prolonged treatment with TMZ did not significantly affect tumor growth based on the tumor size (Fig. 6a) and tumor weight (Fig. 6b).

Taken together, PAMs or PAMs in combination with TMZ significantly reduced tumor size and weight (* p < 0.05, Fig. 6a and b). Although, combined treatment had the same effect as PAMs alone; but the survival was increased significantly than TMZ or PAMs alone (Fig. 6c). MAO A activity was also inhibited by PAMs alone or in combination with TMZ (Fig. 6d). PAMs decreased tumor growth and prolonged the survival of mice bearing invasive glioma brain tumors. This finding suggests that PAMs reduces tumor growth and increases survival in vivo. In these experiments, we used non-toxic low dose TMZ (1 mg/kg), which by itself has no significant effect on tumor size (Fig. 6a) or weight (Fig. 6b).

## Discussion

Increased MAO A has been reported in several types of solid tumors including prostate cancer [1, 16], glioma [2], renal carcinoma [14] and lymphoma [3]. MAO A inhibitors have been shown to reduce glioma in both cell and animal models [2]. MAO A inhibitors and its conjugate reduced the prostate cancer progression and metastasis [1], and suppressed cell growth in glioma [2] and Hodgkin lymphoma [3]. These work underscore the significance of targeting MAO A for cancer therapy.

The natural plant antimicrobial solution PAMs, which contains multi-bioactive components extracted from Chinese natural and folk medicinal plants has been used clinically in China for hundreds of years. Its effective formula has been shown to prevent inflammation via NF-κB signaling pathways. Recent work showed that PAMs inhibits TNF-α/IFN-γ-induced inflammatory cytokines production and inhibits the translocation of p65 in NF-κB signaling pathways [12, 17, 18].

The tumor microenvironment of glioma is largely comprised of inflammatory molecules that affect neoplastic process, proliferation, survival and migration of tumors through inflammation and oxidative stress pathways [19]. It has been suggested that NF-κB activation in cancers may be the result of either exposure to pro-inflammatory stimuli in the tumor microenvironment or mutational activation of upstream components in IκK-NF-κB signaling pathways [17]. It provided evidence that NF-κB is a key transcription factor for the proliferation and survival of glioma cells [20, 21].

This study shows the potential use of PAMs for the treatment of glioblastoma via MAO inhibition.

Multiple functions of PAMS have been reported. We have shown previously the antimicrobial effects of PAMs, on *Pseudomonas aeruginosa*, *Staphylococcus aureus*, *Escherichia. coli, Canidia Albicans,* and *Aspergillus niger* [22]. PAMs remarkably inhibits the growth of *Staphylococcus aureus* and enhance the wound-healing by increasing the permeability of bacterial cell membranes, leakage of contents, and eventually the death of *Staphylococcus aureus.*

PAMs reduced liver cancer growth by regulating apoptosis in HepG2 cells [23]. Also, we showed the anti-cancer effect of PAMs in leukemia cells was mediated by anti-proliferation [24]. Recently, we eported that PAMs could also inhibit the tumor growth of cancers by downregulating the expressions of inflammation and vascular growth associated with TNF-α and VEGF [12].

Our studies showed that treatment with MAO A inhibitor increased TNF-α positive population in tumors from animal models [2]. Recently, it has been reported that treatment with MAO A inhibitor reduced the expression of the oncogene NF-κB in prostate cancer [25–28]. These findings suggest that MAO A inhibitors regulate the inflammatory response to suppress tumor progression [2], and led us to investigate if PAMs may affect MAO A activity.

Here, we demonstrate for the first time that PAMs inhibits MAO A catalytic activity. Further, PAMs reduced the growth of human glioma TMZ-sensitive U251S, TMZ-resistant U251R and mouse glioma GL-26 cells based on MTS, colony formation, and cell migration assays.

Our data shows the IC50 values of PAMs for the growth of U251S, U251R, and GL-26 cells are 115.1, 115.0, 102.4 μg/ml respectively, were 2-fold better than that of clorgyline in all three cell lines. This suggests that herbal medicine mixture PAMs may have more active compounds. The effects of PAMs on normal glia cells are currently under investigation .

TMZ is the current standard therapy for Glioma patients. At least 50% of TMZ treated patients resistant to TMZ. The animal experiments were performed using subcutaneously implanted GL-26 glioma cells in mice. Treatment with PAMs significantly inhibited tumor size, similar to the MAO A specific inhibitor clorgyline. PAMs was also used to treat C57BL/6 mice after they were intracranially implanted with GL-26 glioma cells. The result showed that PAMs significantly inhibits GL- 26 tumor growth in their size and weight (~ 57%). Compared with the vehicle group, PAMs reduced tumor MAO A activity by 25% and improved survival tsignificantly.

We also demonstrated that combining PAMs with low dose of current standard treatment of TMZ was more effective. For the in vivo study, low dose TMZ (1 mg/kg) was used which was lower than the dose administered to patients, non-toxic [2], and no effect on the treatment of glioma. Combining PAMs with low dose TMZ decreased tumor MAO A activity to a greater extent than PAMs or TMZ alone. More importantly, the combination treatment increased survival significantly than either agent alone. TMZ alone had no effect on survival. Thus, PAMs enhances the efficacy of non-toxic low doses of TMZ. This is consistent with our previous finding that MAO A inhibitor clorgyline or the near-infrared-dye conjugated clorgyline (NMI) in combination with TMZ inhibits tumor growth more effectively than each agent alone [2].

Taken together, our findings suggest that PAMs inhibits MAO A activity, has greater effect in combination with non-toxic dose TMZ against glioma, and improves survival in vivo*.* This finding is consistent with our previous studies showing that knock-down (KD) or pharmacological inhibition of MAO A in prostate cancer and glioma reduces cancer progression [1, 2]. Hence, the results show PAMs inhibits MAO A activity and may be used for glioma treatment.

## Conclusions

This is the first study showing that the natural plant antimicrobial solution PAMs has MAO A inhibitory effect and suppresses glioma progression. PAMs has been used to treat skin inflammatory diseases and has effect on pain-releasing and wound healing. Here, we show the potential use of PAMs in combination ttherapy with non-toxic dose of TMZ for drug-sensitive and drug-resistant gliomas.

## Data Availability

All data generated or analyzed during this study are included in this published article.

## Abbreviations

MAO A: Monoamine oxidase A
GBM: Glioblastoma
TMZ: Temozolomide
PAMs: Natural plant antimicrobial solution
IN: Intranasal
IACUC: Institutional Animal Care and Use Committee
IC50: 50% inhibitory concentration

## References

[1] Wu JB, Shao C, Li X, Li Q, Hu P, Shi C, Li Y, Chen YT, Yin F, Liao CP (2014). Monoamine oxidase a mediates prostate tumorigenesis and cancer metastasis. J Clin Invest.

[2] Kushal S, Wang W, Vaikari VP, Kota R, Chen K, Yeh TS, Jhaveri N, Groshen SL, Olenyuk BZ, Chen TC (2016). Monoamine oxidase A (MAO A) inhibitors decrease glioma progression. Oncotarget.

[3] Li PC, Siddiqi IN, Mottok A, Loo EY, Wu CH, Cozen W, Steidl C, Shih JC (2017). Monoamine oxidase a is highly expressed in classical Hodgkin lymphoma. J Pathol.

[4] Bach AW, Lan NC, Johnson DL, Abell CW, Bembenek ME, Kwan SW, Seeburg PH, Shih JC (1988). cDNA cloning of human liver monoamine oxidase a and B: molecular basis of differences in enzymatic properties. Proc Natl Acad Sci U S A.

[5] Lan NC, Chen CH, Shih JC (1989). Expression of functional human monoamine oxidase a and B cDNAs in mammalian cells. J Neurochem.

[6] Youdim MB, Edmondson D, Tipton KF (2006). The therapeutic potential of monoamine oxidase inhibitors. Nat Rev Neurosci.

[7] Weis SM, Cheresh DA (2011). Tumor angiogenesis: molecular pathways and therapeutic targets. Nat Med.

[8] Davis ME (2016). Glioblastoma: overview of disease and treatment. Clin J Oncol Nurs.

[9] Stupp R, Mason WP, van den Bent MJ, Weller M, Fisher B, Taphoorn MJ, Belanger K, Brandes AA, Marosi C, Bogdahn U (2005). Radiotherapy plus concomitant and adjuvant temozolomide for glioblastoma. N Engl J Med.

[10] Han F, Hu R, Yang H, Liu J, Sui J, Xiang X, Wang F, Chu L, Song S (2016). PTEN gene mutations correlate to poor prognosis in glioma patients: a meta-analysis. OncoTargets and therapy.

[11] Sorrentino BP (2002). Gene therapy to protect haematopoietic cells from cytotoxic cancer drugs. Nat Rev Cancer.

[12] Dou R, Liu Z, Yuan X, Xiangfei D, Bai R, Bi Z, Yang P, Yang Y, Dong Y, Su W (2017). PAMs ameliorates the imiquimod-induced psoriasis-like skin disease in mice by inhibition of translocation of NF-kappaB and production of inflammatory cytokines. PLoS One.

[13] Eslami-Farsani M, Moslehi A, Hatami-Shahmir A (2018). Allantoin improves histopathological evaluations in a rat model of gastritis. Physiol Int.

[14] Hodorova I, Rybarova S, Vecanova J, Solar P, Domorakova I, Adamkov M, Mihalik J (2012). Comparison of expression pattern of monoamine oxidase a with histopathologic subtypes and tumour grade of renal cell carcinoma. Med Sci Monit.

[15] Jhaveri N, Cho H, Torres S, Wang W, Schonthal AH, Petasis NA, Louie SG, Hofman FM, Chen TC (2011). Noscapine inhibits tumor growth in TMZ-resistant gliomas. Cancer Lett.

[16] Wu JB, Lin TP, Gallagher JD, Kushal S, Chung LW, Zhau HE, Olenyuk BZ, Shih JC (2015). Monoamine oxidase a inhibitor-near-infrared dye conjugate reduces prostate tumor growth. J Am Chem Soc.

[17] Conti A, Guli C, La Torre D, Tomasello C, Angileri FF, Aguennouz M (2010). Role of inflammation and oxidative stress mediators in gliomas. Cancers.

[18] Landskron G, De la Fuente M, Thuwajit P, Thuwajit C, Hermoso MA (2014). Chronic inflammation and cytokines in the tumor microenvironment. J Immunol Res.

[19] Hanahan D, Weinberg RA (2000). The hallmarks of cancer. Cell.

[20] Gill JS, Zhu X, Moore MJ, Lu L, Yaszemski MJ, Windebank AJ (2002). Effects of NFkappaB decoy oligonucleotides released from biodegradable polymer microparticles on a glioblastoma cell line. Biomaterials.

[21] Nagai S, Washiyama K, Kurimoto M, Takaku A, Endo S, Kumanishi T (2002). Aberrant nuclear factor-kappaB activity and its participation in the growth of human malignant astrocytoma. J Neurosurg.

[22] Zhen F, Song M, Liu Z, Zhou Y, Li D, Mao CQ (2015). Antimicrobial effects and mechanisms of natural plant antimicrobial solution against Staphylococcus aureus. J Biol.

[23] Yuan X, Qiu JY, Wang SY, Dou R, Xiangfei D, Yang P, Mao CQ (2019). Inhibitory effects and preliminary molecular mechanisms of “natural plant antimicrobial solution” (PAMs) on liver cancer HepG2 cells. J Biol.

[24] Zhou Y, Dou R, Bi Z, Yang Y, Liu Z, Wu Y, Qiu J, Wu C, Xiangfei D, Li D, Mao CQ (2015). Promotion of apoptosis in leukemia K562 cells by natural plant antimicrobial solution (PAMs). J China Pharmaceutical Univ.

[25] Lessard L, Saad F, Le Page C, Diallo JS, Peant B, Delvoye N, Mes-Masson AM (2007). NF-kappaB2 processing and p52 nuclear accumulation after androgenic stimulation of LNCaP prostate cancer cells. Cellular signaling.

[26] Chen F, Yang D, Che X, Wang J, Li X, Zhang Z, Chen X, Song X (2012). Livin mediates tumor cell invasion in the DU-145 cell line via NF-kappaB. Oncol Rep.

[27] Ren D, Yang Q, Dai Y, Guo W, Du H, Song L, Peng X (2017). Oncogenic miR-210-3p promotes prostate cancer cell EMT and bone metastasis via NF-kappaB signaling pathway. Mol Cancer.

[28] Zhang J, Kuang Y, Wang Y, Xu Q, Ren Q (2017). Notch-4 silencing inhibits prostate cancer growth and EMT via the NF-kappaB pathway. Apoptosis.

